# Whole-body clearing, staining and screening of calcium deposits in the mdx mouse model of Duchenne muscular dystrophy

**DOI:** 10.1186/s13395-018-0168-8

**Published:** 2018-07-19

**Authors:** Lukasz Bozycki, Kacper Łukasiewicz, Paweł Matryba, Slawomir Pikula

**Affiliations:** 1 Laboratory of Biochemistry of Lipids, Nencki Institute of Experimental Biology, 3 Pasteur Street, 02-093 Warsaw, Poland; 2 Laboratory of Molecular Basis of Behavior, Nencki Institute of Experimental Biology, 3 Pasteur Street, 02-093 Warsaw, Poland; 3 Laboratory of Neurobiology, Nencki Institute of Experimental Biology, 3 Pasteur Street, 02-093 Warsaw, Poland; 4 Department of Immunology, Medical University of Warsaw, 5 Nielubowicza Street, 02-097 Warsaw, Poland

**Keywords:** Duchenne muscular dystrophy, mdx, Optical tissue clearing, Whole-body clearing, Light-sheet fluorescence microscope, Alizarin red S

## Abstract

**Background:**

Duchenne muscular dystrophy (DMD) is a fatal, X-linked genetic disorder. Although DMD is the most common form of muscular dystrophy, only two FDA-approved drugs were developed to delay its progression. In order to assess therapies for treating DMD, several murine models have recently been introduced. As the wide variety of murine models enlighten mechanisms underlying DMD pathology, the question on how to monitor the progression of the disease within the entire musculoskeletal system still remains to be answered. One considerable approach to monitor such progression is histological evaluation of calcium deposits within muscle biopsies. Although accurate, histology is limited to small tissue area and cannot be utilized to evaluate systemic progression of DMD. Therefore, we aimed to develop a methodology suitable for rapid and high-resolution screening of calcium deposits within the entire murine organism.

**Methods:**

Procedures were performed on adult male C57BL/10-mdx and adult male C57BL mice. Animals were sacrificed, perfused, paraformaldehyde-fixed, and subjected to whole-body clearing using optimized perfusion-based CUBIC protocol. Next, cleared organisms were stained with alizarin red S to visualize calcium deposits and subjected to imaging.

**Results:**

Study revealed presence of calcium deposits within degenerated muscles of the entire C57BL/10-mdx mouse organism. Calcified deposits were observed within skeletal muscles of the forelimb, diaphragm, lumbar region, pelvic region, and hindlimb. Calcified deposits found in quadriceps femoris, triceps brachii, and spinalis pars lumborum were characterized. Analysis of cumulative frequency distribution showed different distribution characteristics of calcified deposits in quadriceps femoris muscle in comparison to triceps brachii and spinalis pars lumborum muscles (*p* < 0.001) and quadriceps femoris vs spinalis pars lumborum (*p* < 0.001). Differences between the number of calcified deposits in selected muscles, their volume, and average volume were statistically significant.

**Conclusions:**

In aggregate, we present new methodology to monitor calcium deposits in situ in the mouse model of Duchenne muscular dystrophy. Sample imaging with the presented setup is feasible and applicable for whole-organ/body imaging. Accompanied by the development of custom-made LSFM apparatus, it allows targeted and precise characterization of calcium deposits in cleared muscles. Hence, presented approach might be broadly utilized to monitor degree to which muscles of the entire organism are affected by the necrosis and how is it altered by the treatment or physical activity of the animal. We believe that this would be a valuable tool for studying organs alternations in a wide group of animal models of muscle dystrophy and bone-oriented diseases.

**Electronic supplementary material:**

The online version of this article (10.1186/s13395-018-0168-8) contains supplementary material, which is available to authorized users.

## Background

Recent development of fluorescence-friendly tissue optical clearing (TOC) methods has paved the way for imaging of large, non-sectioned specimens [1]. Rendering specimens transparent not only protects the tissue from deformation during sectioning, such as tearing, folding, stretching etc., but also helps to avoid under sampling and distortion during three-dimensional volumetric reconstruction. Originally, TOC methods were developed to clear ~ 1-mm-thick murine brain slices. Within few years, the methodology and its application expanded, making it possible to render transparent whole brains, peripheral organs, and even whole bodies of rodents. This, in turn, set foundations for organism-level systems biology in which particular processes might be studied simultaneously within the entire organism [2, 3].

Recently, light-sheet fluorescence microscopy (LSFM), also known as selective plane illumination microscopy (SPIM), has been developed to visualize naturally transparent specimens, such as zebrafish embryos and *Caenorhabditis elegans* [4]. Consequently, in LSFM, the sample is illuminated by a thin sheet of fluorescence excitation light and the emission light passes through the lens perpendicular to the camera. As long as the specimen is translucent, plane by plane scanning is feasible and allows for fast generation of 3D images with minimal photobleaching and phototoxicity, when compared to confocal and wide-field fluorescence microscopy [5–7]. Development of sophisticated TOC approaches stimulated construction of LSFM setups adapted for specific needs, variety of refractive indices of tissues, and matching media [8, 9]. Nonetheless, while methods for rendering the entire rodent bodies transparent have been developed, their imaging is prevented by the optics and size of the imaging chambers of commercially available LSFM.

Duchenne muscular dystrophy (DMD) is a lethal X-chromosome-linked recessive disorder, affecting 1 in 3500–5000 male births [10], caused by various mutations in a gene encoding a membrane-associated protein—dystrophin [11]. Loss of dystrophin function leads to instability of muscle fibers, which undergo repeated cycles of degeneration and regeneration, and finally to severe muscle wasting. One of the prominent symptoms of DMD is compromised function of sarco/endoplasmic reticulum Ca^2+^ ATPase (SERCA) and of several Ca^2+^ ion channels that results in impaired Ca^2+^ homeostasis which, in turn, activates cytosolic proteases and leads to necrosis and formation of numerous calcium deposits [12]. Calcium deposits develop as this fatal disorder progresses, contributing to respiratory and/or cardiac muscle weakness and death. Therefore, many studies aimed at characterizing such deposits to improve our understanding of the molecular mechanisms of DMD development and find ways to stop its progression [13–16].

As DMD affects skeletal muscles of the entire organism at various time points and to various degrees, it is desirable but challenging, to localize and quantify calcium deposits (calcium-positive muscle fibers) in the entire musculoskeletal system. Calcium deposits serve as a histological hallmark of DMD but not obviously of other myopathies, e.g., myotonic dystrophy type 1 [17]. Until now, analyses were performed either histologically or with gross anatomy imaging techniques such as magnetic resonance imaging (MRI), computed tomography (CT), or recently optical coherence tomography [18–21]. While histological sectioning offers highly detailed examination of single slices, it seems vastly time- and labor-consuming, not suitable for performing comprehensive analyses on the entire organs/organism. On the contrary, CT/MRI devoted to visualize gross anatomy does not reach the resolution required to visualize discrete sites harboring calcium deposits [22, 23].

Therefore, we endeavored to utilize the concept of whole-body clearing to be able for the first time to localize calcium deposits in the entire body of mdx mouse (C57BL/10-mdx) [24]. Although mdx mouse model is known to exhibit a much milder phenotype compared to DMD patients, it is the most commonly used model in DMD studies and evaluation of potential therapeutics, such as VBP15, arginine pyruvate, or P2RX7 antagonists [25, 26]. Successful clearing and development of a calcium staining protocol presented us with the challenge of visualizing such large, transparent specimens.

**Fig. 1:**
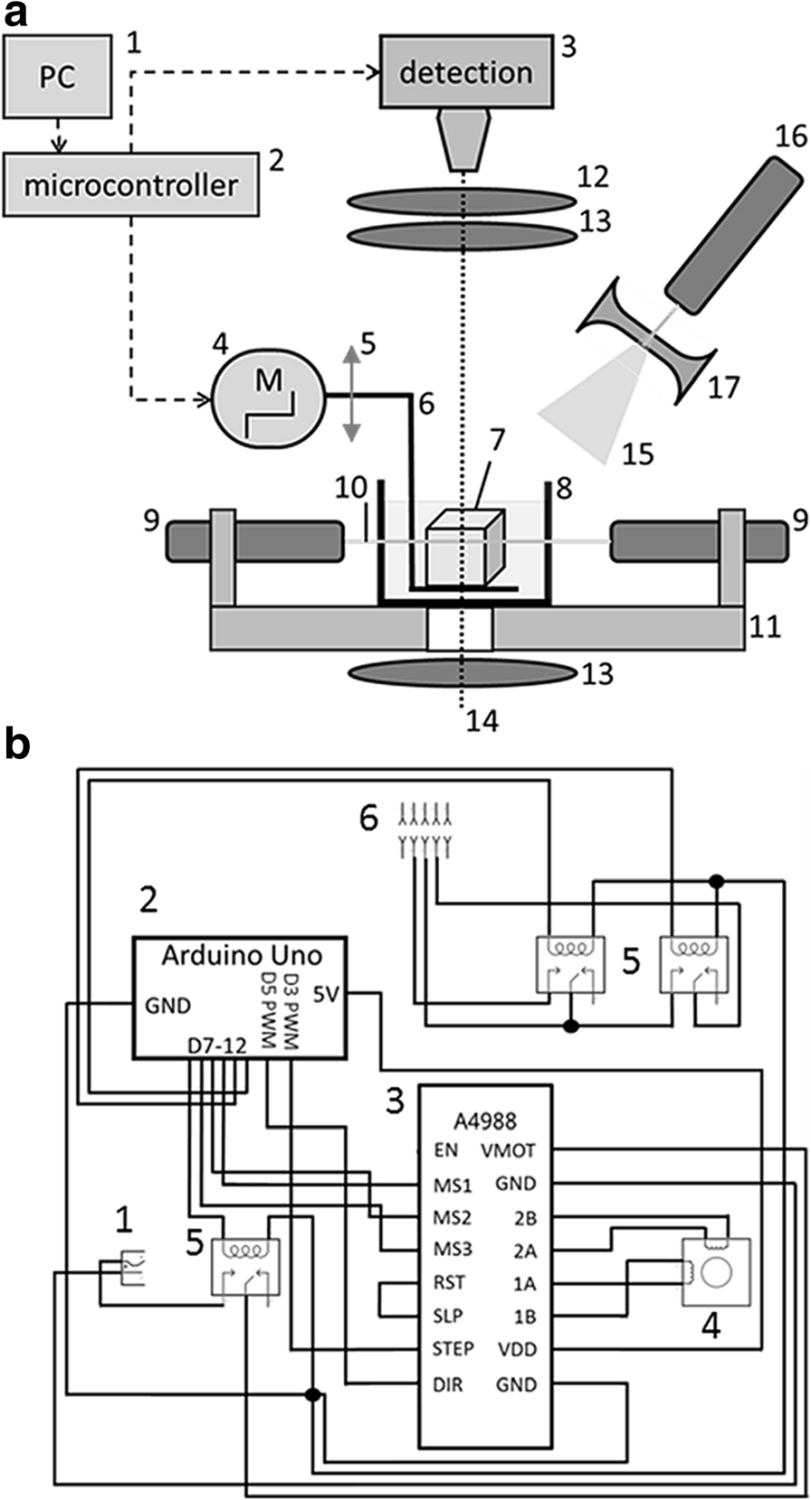
Apparatus for macroscopic imaging using light-sheet illumination. **a** Device overview. Scanning sequence is configured on computer (1) with software dedicated for microcontroller (2). Microcontroller controls the sequence of image capturing by the detection unit (3) and movement of the stepper motor (4). Stepper motor is attached to the z-axis line module (5) connected with base holder (6). Specimen (7) is placed on a base holder and put inside glass container (8). Laser light is emitted from lasers placed on both sides of the sample (9) to form one plane (10) perpendicular to the optical axis (dotted line). Lasers are placed in a metal block to provide cooling and stabilization (11). A filter set (12) is placed on the optical pathway to remove excitation, crossed polarizers (13) and light emitting diode light (14). To acquire diverged laser light (15), we used laser (16) with negative lens (17). **b** Technical scheme. Schematic view of connections between power supply (1), microcontroller (Arduino UNO) (2), stepper motor driver (A4988) (3), stepper motor (4), relays (5), and camera control (6)

In view of the lack of proper LSFM to quickly image large transparent specimens and the cost of the existing setups, we intended to develop a system for such large-scale imaging. Thus, we have built an inexpensive dual-sided illumination apparatus based on LSFM general designs (Fig. 1). Our custom-made setup is compatible with various staining chromophores and enables straightforward reconstruction of anatomically relevant structures of rodent organs such as heart or cerebellum. Together with optimized tissue clearing and staining technique, we present new methodology for precise localization and quantification of calcium deposits within the soft tissues of the entire mdx mouse model of DMD.

## Methods

### Animals

Procedures were performed on three male Wistar rats (8 weeks old) and adult 8-week-old male mice: six dystrophic (57BL/10ScSn-Dmd<mdx>/J) and six control (C57BL/10ScSnJ, Jackson Laboratory). All animal procedures were performed according to the Polish law and EU directive (Directive 2010/63/EU).

### Animal perfusion

Mice (mean body weight = 20 g ± 2) and rat (body weight = 180 g), were deeply anesthetized with intraperitoneal injection of lethal dose of sodium pentobarbital (100 mg/kg). Then, animals were perfused transcardially with 100 and 200 ml, respectively, of 0.1 M ice-cold phosphate-buffered saline (PBS). To further increase efficiency of perfusion, heparin (5000 IU/ml, final concentration of 0.1% *v*/*v*) was added to PBS. In the next step, animals were perfused with 100 and 200 ml, respectively, of freshly prepared 4% paraformaldehyde (PFA) in PBS. Animal skin was then gently removed under the fume-hood with scalpel and scissors.

### Whole-body tissue clearing

Immediately following standard cardiac perfusion (see the “Animal perfusion” section), animals were further perfused with 100% reagent-1 (R1) prepared according to the *Clear*, Unobstructed Brain/Body Imaging Cocktails and Computational analysis (CUBIC) protocol as described by Susaki and co-workers [27]. R1 consists of 25 wt% urea (#U5378, Sigma), 25 wt% *N*,*N*,*N*′,*N*′-tetrakis(2-hydroxypropyl) ethylenediamine (#122262, Sigma), and 15 wt% Triton X-100 (#X-100, Sigma). R1 perfusion was performed according to perfusion-based CUBIC protocol as described previously [28]. Briefly, R1 was perfused for 2–3 days with a pumping speed of 5 ml/min. Three hundred milliliters of R1 was refreshed twice, every 24 h. Next, rat organs were dissected and placed in 20 ml of R1 each, in polypropylene containers. Rat organs were then kept for the next 1–2 days in 37 °C with gentle shaking. Three Wistar rats, 4 C57BL/10-mdx, and 4 C57BL/10 mice were subjected to whole-body clearing and staining in total.

### Propidium iodide staining

Propidium iodide (#P21493 LifeTechnologies) at concentration of 7.5 μg/ml can be added at any point during the perfusion-based clearing and dissolved directly in R1 solution. Single dose of propidium iodide (dissolved in 300 ml of R1) was sufficient to stain every organ.

### Alizarin red S staining

Following clearing, solution was replaced with 300 ml of R1 solution with alizarin red S, commonly used for demonstration of calcium salts [29], at final concentration of 0.03%. The next day, the solution was again exchanged with 300 ml of R1 to remove the excess of unbound alizarin red S.

### Histology

Samples from two mdx and two control mice were collected, washed with 0.1 M PBS twice for 5 min and fixed with 4% PFA for 24 h in 4 °C. Samples were than washed with 0.1 M PBS, subjected to autotechnicon tissue processor and sliced for 4 μm. Next, slices were subjected to standard hematoxylin and eosin histological staining or deparaffinized using following protocol: heating in 55 °C (10 min), incubation in xylene (10 min), xylene to ethanol 1:1 (5 min), 99,8% ethanol (3 min), 70% ethanol (3 min), 30% ethanol (3 min), distilled water (3 min), and subjected to 2% *w*/*v* alizarin red S solution (30 s) followed by wash in running water (30 s).

### Device setting

Electronic elements of the equipment were connected according to the technical scheme shown in Fig. 1b. Arduino software (Arduino 1.6.12) was used to set the sequence of (1) turning on the power supply, (2) moving a defined number of steps by the stepper motor, and (3) triggering image acquisition. Sequence was uploaded to Arduino microcontroller (Arduino UNO). For image detection, we used a digital camera (Nikon D7100) equipped with objectives mounted with a reverse ring adapter (nikkor AF-S 70-200 f/4 G ED VR, nikkor AF-S 50 mm f/1.8G) and filter (Midopt Fil BP635/58) to remove the emission light. The microcontroller was connected with a detection unit via two relays as shown in Fig. 1b, to control the camera shutter and buffer. The microcontroller was connected with a bipolar stepper motor (JK42HS48-1204 with 200 steps/rotation 1.8°) via a driver to ensure precise movement control (A4988, which provides movement at 1/16 step resolution). The stepper motor was connected with a *z*-axis line module via a clutch. Here, we used light microscope body (Zeiss), which determined *z*-axis step resolution to 5.5 μm but any type of microscope focusing block which provide precise *z*-axis movement could be used instead. Metal holder was connected with the line module and served as the base for mounting the specimen (Fig. 1a6). During imaging, both base and the specimen were submerged in the buffer inside a glass container (Fig. 1a8, custom made). Laser light was emitted from line lasers (Fig. 1a9 532 nm and 10 mW, composed of basic pointer laser and 100 nm optical fiber used as cylindrical lens) placed on both sides of the sample and calibrated to form one plane of light perpendicular to optical axis. Both lasers were attached to a custom made aluminum block with thermal glue to provide cooling and stabilization. Cross polarization images were acquired using two crossed line polarizers (MidOpt) and white light emitting diode (3 W white LED).

### Image acquisition, computer, and statistical analysis

**Fig. 2:**
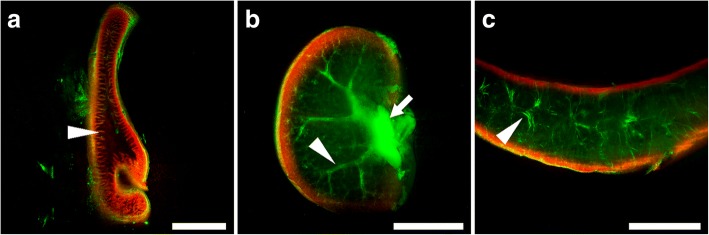
Light-sheet sections of perfusion-based CUBIC cleared rat organs. Stained with propidium iodide (red) and anatomical structures visible in scattered laser light (green). **a** Small intestine with villi (arrowhead). **b** Kidney with renal pelvis (arrow) and minor calyx (arrowhead). **c** Spleen with trabeculae (arrowhead). Scale bar, 5 mm

**Fig. 3:**
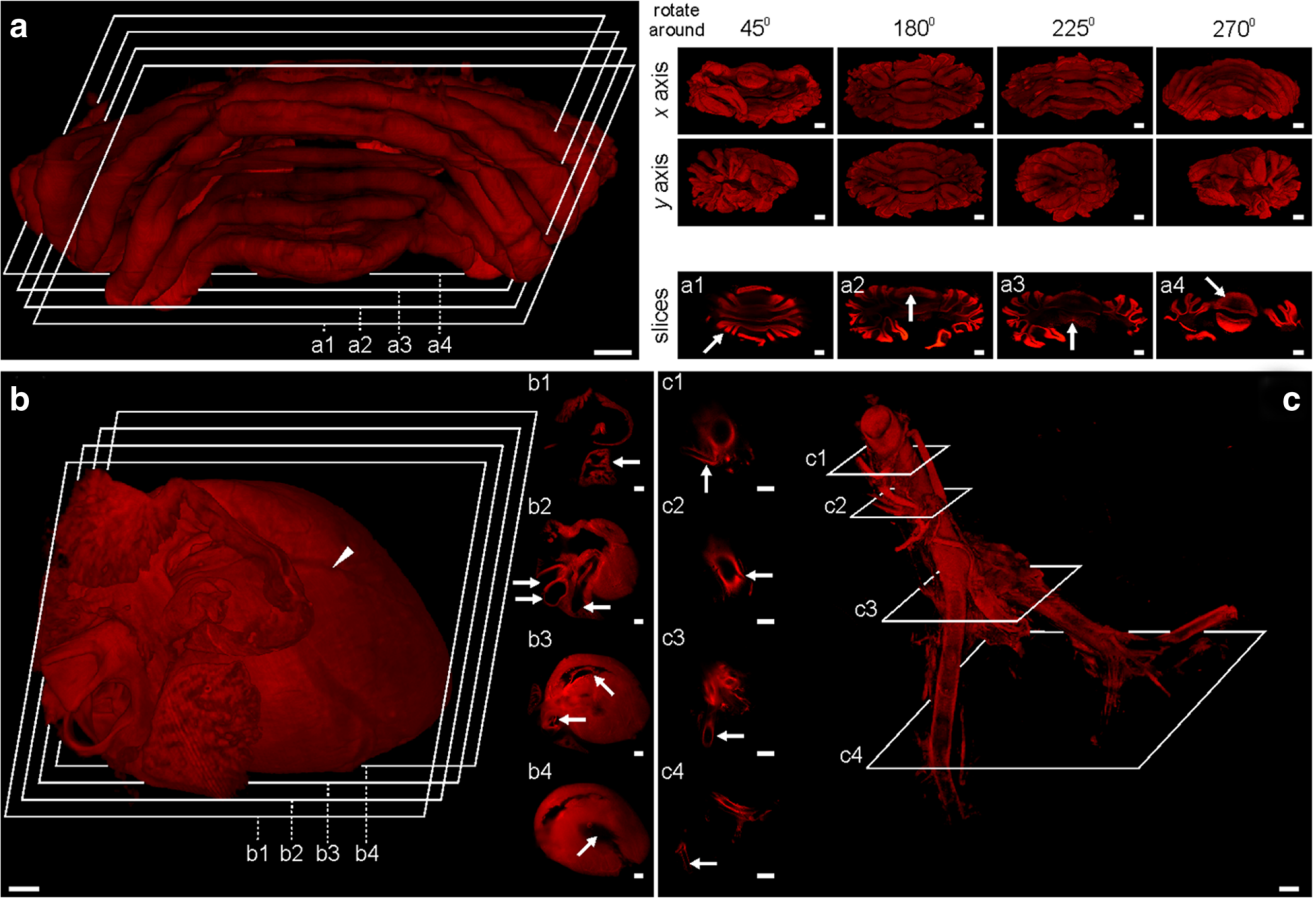
Three-dimensional reconstructions of perfusion-based CUBIC cleared rat organs. **a** Cerebellum, 3D view from different angles and (a1–a4) single slices. Single sections represent foil of cerebellum (a1), vermis (a2), medullary velum (a3), and cerebellar peduncle (a4). **b** Heart reconstruction presenting the auricles, aorta, and coronary vessels (arrowhead). Single sections represent the auricle (b1), great arteries (b2), mitral and pulmonary valve (b3), and left ventricle (b4). **c** Abdominal aorta bifurcation into the left and right common iliac arteries. Single sections represent the arteries: renal (c1), inferior mesenteric (c2), common iliac (c3), and external iliac (c4). Scale bar, 1 mm

**Fig. 4:**
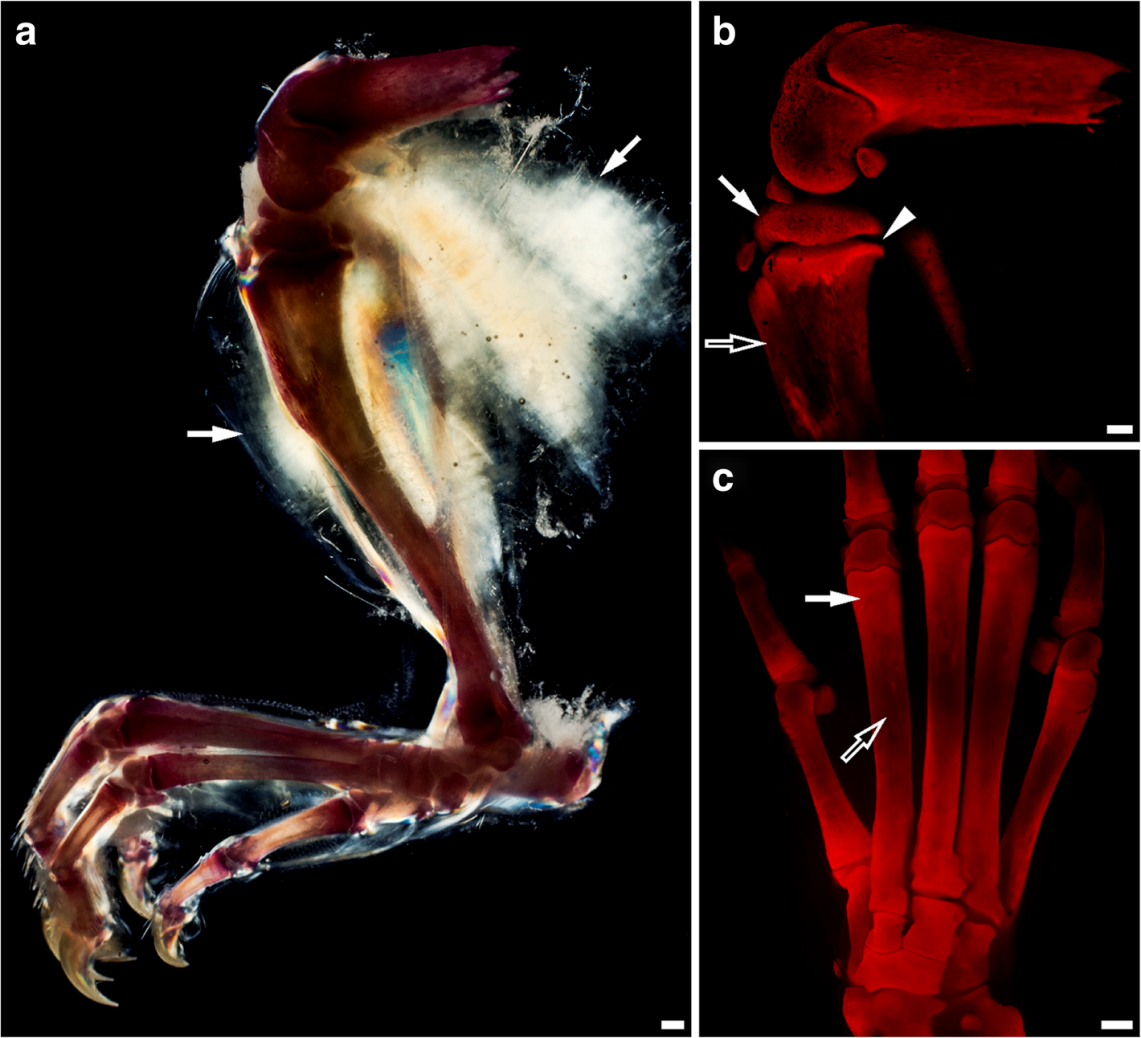
Rat hindlimb stained with alizarin red S. **a** Cross polarization image of hindlimb bones visible through clarified soft tissue (arrows). **b** Fluorescent image of magnified knee joint with spongy (full arrow) or compact (empty arrow) bone and epiphyseal line (arrowhead). **c** Metatarsal area with visible difference in bone density of metaphysis (full arrow) and diaphysis (empty arrow). Scale bar, 1 mm

**Fig. 5:**
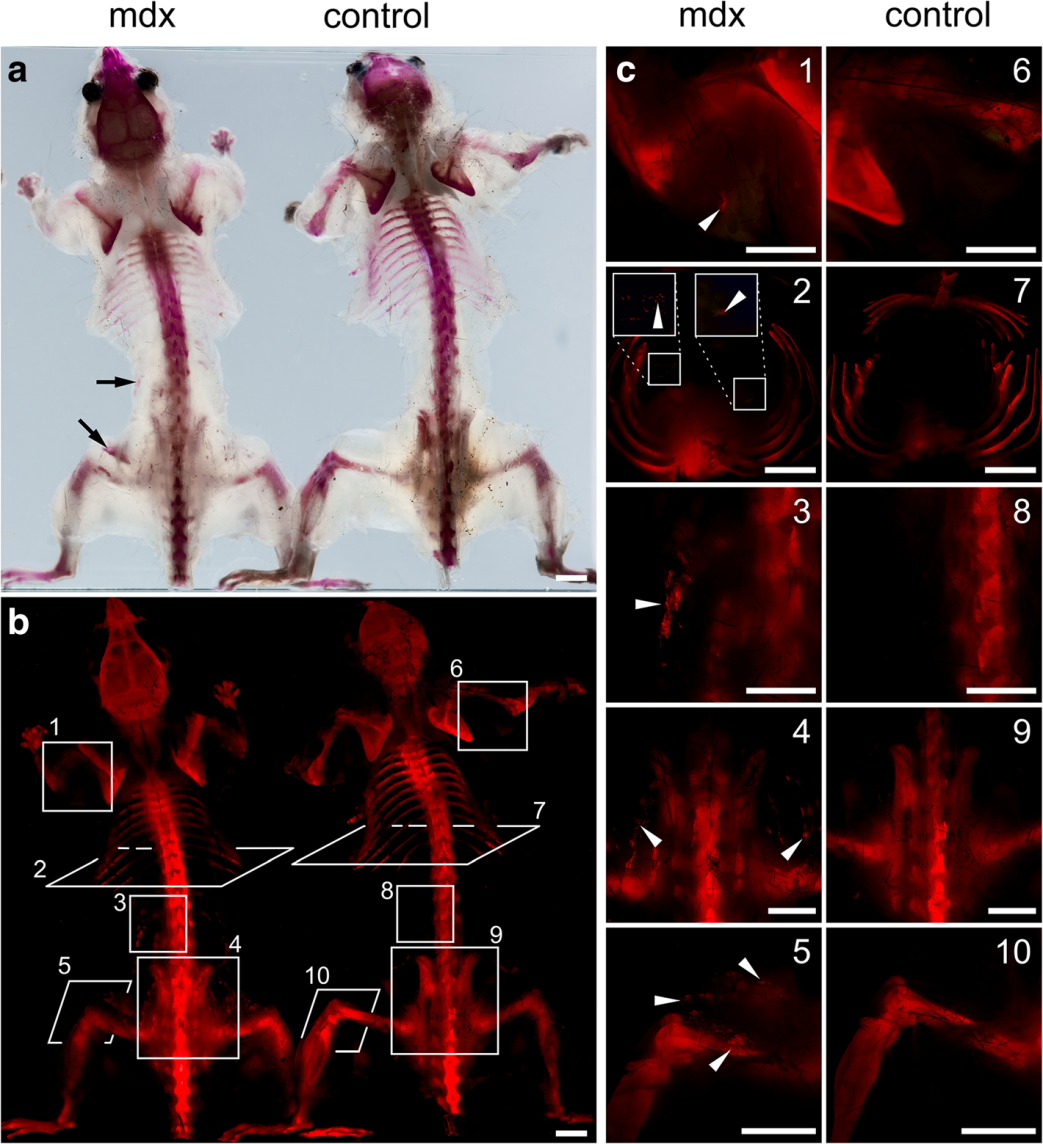
Comparative imaging of whole-body cleared mice. **a** Bright-field images (dorsal view) of perfusion-based CUBIC-cleared mdx model of DMD and control mouse stained with alizarin red S. Visible skeleton and calcifications within muscles (arrows). **b** Fluorescent image of mice with visible skeleton, calcifications within muscles and selected planes (1–10) in **c** magnified views. **c** Arrowheads indicate clusters of pathological calcium deposits in forelimb (1, 6), diaphragm (2, 7), lumbar region (3, 8), pelvic region (4, 9), and hindlimb (5, 10). Scale bar, 1 cm

**Fig. 6:**
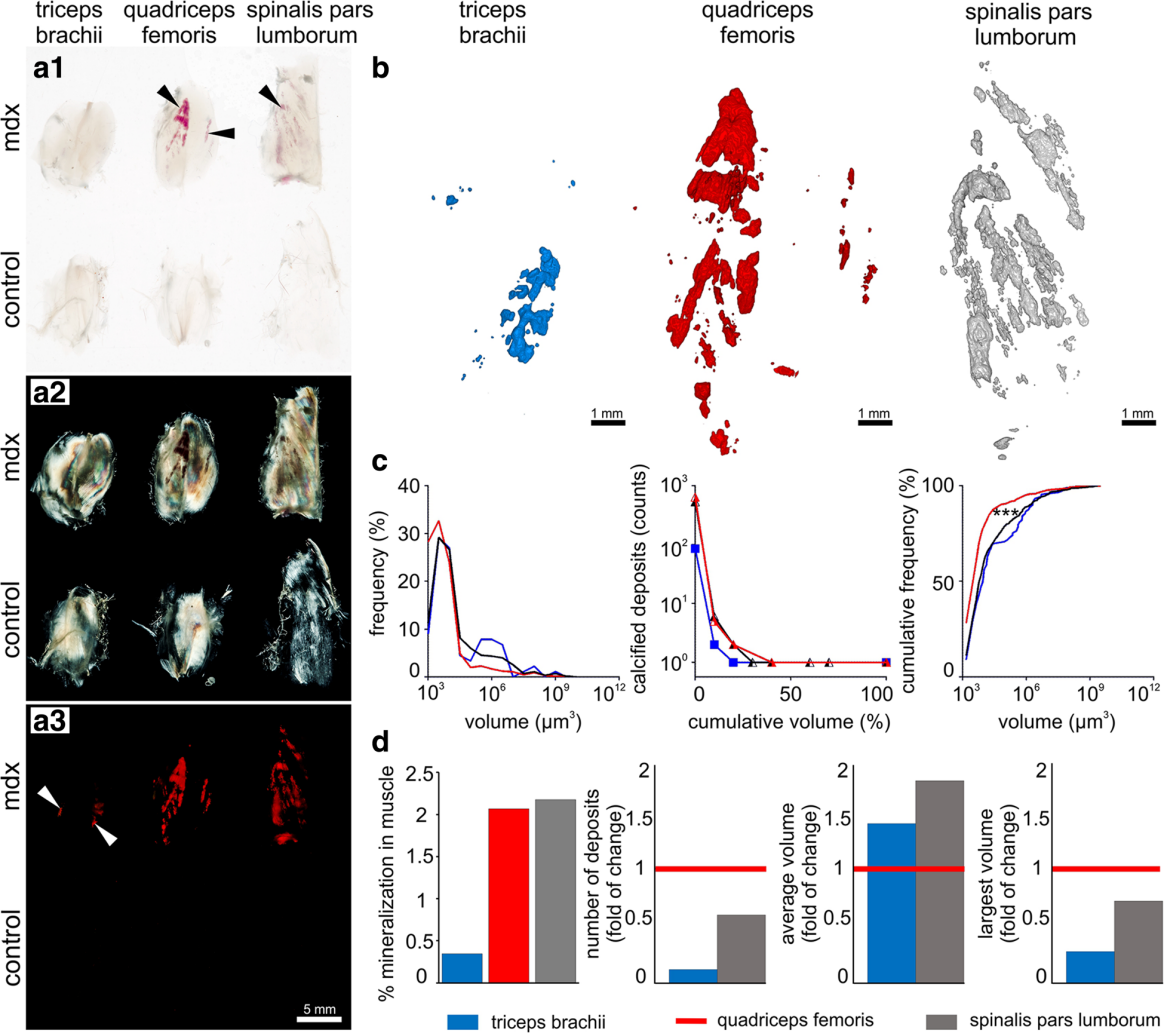
Comparative imaging of calcified deposits in mdx and control mice muscles. Triceps brachii, quadriceps femoris, and spinalis pars lumborum muscles were dissected from perfusion-based CUBIC-cleared mdx model of DMD and control mouse, stained with alizarin red S and imaged in bright-field (**a1**), crossed polarized light (**a2**), and fluorescence (**a3**). Arrowheads indicate calcified deposits. **b** Three-dimensional light-sheet reconstructions of calcified deposits in muscles of mdx mouse. **c** Quantitative volume analysis of frequency distribution (left), relationship between cumulative volume and counts of calcified deposits (middle), cumulative frequency distribution (right) in triceps brachii (blue line and squares), quadriceps femoris (red line and triangles), and spinalis pars lumborum (black line and triangles). Two-sample Kolmogorov-Smirnov test, *p < 0.001 quadriceps femoris vs triceps brachii, quadriceps femoris vs spinalis pars lumborum. **d** Comparative volume analysis of calcified deposits in muscles of mdx mouse normalized to quadriceps femoris. Number of calcified deposits in mdx mouse shown in **b**, **c**, **d**: triceps brachii (*n* = 89), quadriceps femoris (*n* = 652), spinalis pars lumborum (*n* = 548)

Photos (Figs. 2, 3, 4, 5, and 6) were taken with a resolution of 6000 × 4000 px. The image format was .jpeg. Figures were made in CorelDRAW X6, Photoshop CS6, and GraphPad Prism. 3D reconstructions of the images were performed at 16.5 μm step in the *z*-axis. 3D reconstructions of organs (Fig. 3) and calcified deposits (Fig. 6) were obtained using the Fiji (ImageJ1.50i, NIH); 3D Viewer plugin, and 8-bit images were used. For the determination of the amount of calcified deposits Measure function in the Fiji program was used. Calcified deposits were photographed using a filter (Midopt Fil BP635/58). No filter image was used to determine total muscle volume. GraphPad Prism was used for statistical analysis. For comparison of the distributions, the two-sample Kolmogorov-Smirnov test was used [30].

## Results

### Development of experimental setup

Variety of experimental designs and chemically different TOC techniques stimulated development of several commercial and custom-made LSFM setups presented recently [8, 9, 31]. Following the general designs of LSFM, we aimed at simplifying the setup, making it a much less expensive, yet powerful, approach enabling precise imaging of large, transparent samples, possibly the entire mouse bodies.

To fulfill this goal, we constructed our own device (Fig. 1a and Additional file 1: Figure S1). We used a base body stand of a light microscope (Zeiss), which provided precise movement in *z*-axis when connected with a stepper motor. Sample was illuminated either with two line lasers placed opposite to each other and forming one light plane or with diverged laser light. Dual-sided illumination is indispensable when imaging large specimens (e.g., rat hindlimb), as it guarantees even illumination. Our device was controlled by a simple programmable microcontroller. The microcontroller was connected with the *z*-axis module and camera via a circuit presented in Fig. 1b. The specimen was placed on a home-made holder attached to the *z*-axis module. Movement of the sample provided plane change with fixed camera focus while the cuvette was immobile. To obtain whole-sample images, we used crossed-polarized light or diverged laser light.

### Imaging of cleared organs with custom-made apparatus facilitates volumetric reconstruction

By establishing perfusion-based CUBIC clearing procedure, we were able to effectively make transparent not only mouse but also rat bodies within 3 and 4 days, respectively, and thus introduced the latter to whole-body imaging. However, the size of rat organs limits their imaging performance with commercially available LSFMs. Therefore, in this report, we describe examples of using our device to analyze rat samples. As the first, we show light-sheet sections of various rat organs stained with nuclear dye—propidium iodide (Fig. 2 and Additional file 1: Figure S2). Captured sections present details such as intestinal villi, renal pelvis, and minor calyx in kidney or trabeculae in spleen. Scattered laser light facilitated detection of macroanatomical structures. Next, we performed 3D volumetric reconstructions of rat organs using light-sheet sections, acquired from top to the bottom (Fig. 3). Reconstructed cerebellum consists of 448 sections (Fig. 3a), heart consists of 762 sections (Fig. 3b), and aorta of 295 sections (Fig. 3c). 3D volumetric reconstructions allowed for unrestricted rotation of visualized organs as it is presented for cerebellum. Such type of data presentation exposed macroanatomical structures in reciprocal relationship, spatial arrangement and allowed us to identify, e.g., folia of cerebellum (Fig. 3a1), vermis (Fig. 3a2), medullary velum (Fig. 3a3), cerebellar peduncle (Fig. 3a4) in the cerebellum; auricle (Fig. 3b1), great arteries (Fig. 3b2), mitral and pulmonary valve (Fig. 3b3), left ventricle (Fig. 3b4) in the heart; renal (Fig. 3c1), inferior mesenteric (Fig. 3c2), common iliac (Fig. 3c3), external iliac (Fig. 3c4) arteries in the aorta.

### Custom-made setup is compatible with various staining dyes and enables analysis of large specimens

Taking advantage of the fact that the upper sample size limit to be imaged by our apparatus is defined solely by the size of the imaging chamber (and laser line sharpness, which decreases when moving from beam waist), we endeavored to visualize the entire adult rat hindlimb skeleton. After screening, we decided to proceed with alizarin red S, a broadly used dye for detection of calcium deposits [32]. Thus, we elaborated a protocol suitable for performing such staining with water-based cleared tissues distinct from histological sections. The successful protocol allowed us to obtain detailed in situ images of the entire skeleton of rat hindlimb (Fig. 4a). Imaging revealed the anticipated sites of gradual change of signal intensity associated with different bone density, such as spongy and compact bone, epiphyseal line, metaphysis, or diaphysis (Fig. 4b, c).

### Whole-body mouse skeleton and pathological calcium deposits imaging

Having optimized the protocol for calcium deposit staining, we decided to analyze the whole-body clearing of mdx DMD mice. Notably, after successful whole-body clearing, we were able to perform imaging of two mouse bodies simultaneously (mdx and control). Bright-field images demonstrate that the staining intensity and transparency of these two species are similar. Calcium deposits in skeletal muscles are present in mdx but not control mouse (Fig. 5a and Additional file 1: Figure S3). We diverged the laser light using a negative lens to obtain images of the whole murine skeleton. We observed calcified deposits within mdx mouse skeletal muscles of the forelimb, diaphragm, lumbar region, pelvic region, and hindlimb (Fig. 5b, c). This result allowed us to perform targeted muscle group selection for quantitative analysis. In order to screen for pathology in various body regions, we have chosen muscles involved in forelimb, hindlimb, and spine movements. We dissected samples from the area of triceps brachii, quadriceps femoris, and spinalis pars lumborum muscles from mdx and control mice. Samples visualized in bright-field revealed the presence of calcified deposits in quadriceps femoris and spinalis pars lumborum in mdx, but not control mouse (Fig. 6a1). Images obtained with crossed polarized light confirmed that muscle volumes were comparable (Fig. 6a2). Fluorescent images exposed previously observed calcified deposits as well as deposits imperceptible in bright-field images within triceps brachii and further confirmed their absence in control muscles (Fig. 6a3). Based on these observations, we made three-dimensional light-sheet reconstructions of calcified deposits from 413 sections of triceps brachii, 339 sections of quadriceps femoris and 409 sections of spinalis pars lumborum muscles (Fig. 6b). Image post-processing in Fiji enabled us to compare distribution of calcium deposits between the studied groups (Fig. 6c and Additional file 1: Figure S4). We observed that in triceps brachii calcified deposits are characterized by bimodal frequency distribution in contrast to those found in quadriceps femoris and spinalis pars lumborum muscles (Fig. 6c, left). Cumulative volume analysis revealed that 83% of total calcified deposit volume is formed by one deposit in triceps brachii. In quadriceps femoris, the biggest calcified deposit accounts for 57% of the total volume, while two calcified deposits account for 78%. In contrast, 27% of total calcified deposit volume is formed by one deposit in spinalis pars lumborum, while five calcified deposits account for 81% (Fig. 6c, middle).

Cumulative frequency distribution analysis showed different distribution of calcified deposits in quadriceps femoris muscle in comparison to triceps brachii and spinalis pars lumborum muscles (Fig. 6c, right; quadriceps femoris vs triceps brachii; Kolmogorov-Smirnov D (652, 89) = 0.280; *p* < 0.001; quadriceps femoris vs spinalis pars lumborum; Kolmogorov-Smirnov D (652, 548) = 0.214; *p* < 0.001; triceps brachii vs spinalis pars lumborum; Kolmogorov-Smirnov D (89, 548) = 0.091; *p* = 0.544). In comparison to other muscles, quadriceps femoris contains many relatively smaller calcified deposits. For further analysis of the calcified deposits of quadriceps femoris in comparison to other muscles, we took into account the muscle volume with quadriceps femoris as a reference. We observed that the total volume of calcification was highest for spinalis pars lumborum and lowest for triceps brachii (Fig. 6d; triceps brachii to quadriceps femoris to spinalis pars lumborum, 0.17:1:1.05). The number of calcified deposits was highest for quadriceps femoris and lowest for triceps brachii (0.12:1:0.59); this trend was correlated and further confirmed by histopathology (Additional file 1: Figure S3). The average volume of calcified deposits was highest for spinalis pars lumborum and lowest for quadriceps femoris (1.39:1:1.77). The volume of calcified deposits was largest for quadriceps femoris and smallest for triceps brachii (0.27:1:0.72).

## Discussion

Tissue optical clearing (TOC) has emerged as a valuable approach, complementary to histology and gross imaging, to study organs in 3D. Recent advancements in TOC resulted in development of whole-body clearing protocols enabling researchers, in theory, to study a particular process within the entire fixed organism [33, 34]. Although all of the three distinct TOC approaches (water-based, solvent-based, and hydrogel-based) have been applied to perfusion-based whole-body clearing, we decided to proceed with CUBIC (a water-based method) for calcium deposits screening for a few reasons. First, it was already reported that CUBIC is capable of clearing mouse muscle tissue fast and effectively and what is prominent, it accomplishes transparency without employment of electric field or controlling of temperature, both of which are potentially harmful for samples processed with CLARITY (hydrogel-based method). Hitherto, two protocols of CLARITY optimized for skeletal muscle clearing were presented [35, 36]. While these might emerge as a considerable alternative in the future, additional attempts should be performed to reduce incubation time (currently above 40 days) and imaging depth. Second, CUBIC is a straightforward technique utilizing only one reagent, made out of four inexpensive ingredients. Markedly, in comparison with BABB, uDISCO (solvent-based method) refractive index matching solution, the CUBIC reagent is non-toxic, which is even more important, while handling huge volumes of reagent, as it the case during whole-body clearing protocols. Finally, it does not alter general histological appearance of muscles (Additional file 1: Figure S5).

Implementation of whole-body clearing to monitor the progression of DMD as represented by accretion of calcium deposits is expedient albeit requires not only successful clearing but also a proper imaging technique [37]. In view of the lack of commercially available LSFM to image entire transparent structures, such as rat hindlimb, we endeavored to develop our own system to be able to (1) overcome the mentioned size limitation, (2) process 3D volumetric reconstructions of calcium deposits in situ, and finally (3) make it feasible to easily rebuild and adjust the system to specific needs. Our custom-made system meets the mentioned criteria. From our perspective, the system is simplified, consisting of line lasers, glass container, specimen, stepper motor to move the specimen in the *z*-axis, filter, and camera which can notably be easily removed and replaced. This means that the system can, potentially, be compatible with every kind of chemical staining in terms of precise adjustment of excitation lasers and narrow-range filters. Moreover, usage of digital camera, the objectives of which could be easily adapted, instead of a system of specialized lenses, not only remarkably reduces the cost but is especially important when imaging solvent-based cleared specimens (e.g., 3DISCO), as solvents are prone to damage most of the available lenses [38]. Here, we could use objectives with a long working distance and avoid immersing them in the media. Therefore, our apparatus allows inexpensive imaging of organs cleared with chemically distinct protocols.

By combining imaging with previously established optimization of CUBIC tissue clearing technique, we were able to put the whole-body clearing concept to practice and screen for sites of calcium deposits in a mdx mouse model of DMD. It has to be reemphasized that treatment of DMD is still limited. Current guidelines suggest glucocorticosteroids, either prednisone or deflazacort, as the first-line therapies [39]. Glucocorticosteroids improve the number of both muscle strength and function parameters (e.g., muscle score, ability to lift weights, time to stand, or four-stair climbing time), and most notably prolong patient time to loss of ambulation [40]. However, such therapy does not bear potential to cure DMD patients and accounts for severe adverse effects, which strengthens the urgent need to discover novel therapies [41, 42]. One possible way to examine the effectiveness of new therapeutics is to measure their ability to stabilize sarcolemma by assessing the number of calcific deposits in treated vs. control animals. Thus far, such analysis was insufficient and performed either in a limited number of histological sections or by gross imaging techniques, such as MRI/μCT. As we have showed, such experiments might be performed in a time- and cost-effective way for large groups of muscles. Notably, histological sectioning stays in line with our sample volumetric analysis, presenting significantly reduced number of calcific deposits between fore- and hindlimb muscles. Hitherto, several hypothesis have been proposed to explain why various muscle groups are affected to different extent in mdx mouse. Factors studied and taken into account are, e.g., (1) muscle fiber type [43], (2) muscle fiber innervations [44], (3) changes in gene expression profile [45], (4) locomotor activity altering calcium homeostasis [46], and (5) work-overload hypothesis [47]. Although presented approach cannot reveal molecular mechanisms underlying observed variety in the degree of dystrophy between limbs, it is reasonable to think it might be convenient to characterize such differences in detail in the course of mdx mouse life and treatment.

Moreover, we show that imaging with our custom-made system grants successful volumetric reconstructions of peripheral mouse/rat organs, making it promising technique for screening of calcified tissues in variety of rodent models. Such reconstructions might be easily implemented to quantify anatomical changes of which detection and analysis do not require cellular resolution but which are imperceptible by gross imaging techniques such as MRI/CT scanners. It is reasonable to believe that presented methodology could be successfully adapted for studies involving other bone-orientated diseases such as osteoporosis, multiple myeloma, osteitis deformans, or even sclerotic plaques in the course of arteriosclerosis.

## Conclusions

In aggregate, we present new methodology to monitor calcium deposits in situ in the mouse model of Duchenne muscular dystrophy. Sample imaging with the presented setup is feasible and applicable for whole-organ/body imaging. Accompanied by the development of custom-made LSFM apparatus, it allows targeted and precise characterization of calcium deposits in cleared muscles. Hence, presented approach might be broadly utilized to monitor degree to which muscles of the entire organism are affected by the necrosis and how is it altered by the treatment or physical activity of the animal. We believe that this would be a valuable tool for studying organ alterations in a wide group of animal models of muscle dystrophy and bone-oriented diseases.

## Additional file

Additional file 1: **Figure S1.** Implementation of apparatus for macroscopic imaging using light-sheet illumination. (a) General view. (b) Side view. (c) Front view. (1) camera, (2) stepper motor, (3) z-axis line module (here light microscope body, Zeiss), (4) base holder, (5) glass container, (6) line lasers, (7) metal block, (8) laser power supply. **Figure S2.** Perfusion-based CUBIC cleared rat organs stained with propidium iodide. Bright field images of whole rat organs (a) intestine, (b) kidney, (c) heart, (d) cerebellum and (e) spleen. Single squares in all panels - 5 × 5 mm. This figure has been adapted from the original article “Optimized perfusion-based CUBIC protocol for the efficient whole-body clearing and imaging of rat organs” by P. Matryba et al., J Biophotonics 2017, doi./10.1002/jbio.201700248. Reproduced with permission 4358370973288. **Figure S3.** Comparison between optical and standard histopathology sectioning of Alizarin red S stained mdx mouse muscles. (a) Representative optical sections acquired during imaging with custom-made LSFM. Control animals present no staining-positive tissue. (b) Representative histopathology 4 μm sections observed with low and (c) high magnification. It has to be noted that histopathology images stay in line with optical sectioning, presenting petite deposits in triceps brachii and similar amount of deposits between quadriceps femoris and spinalis pars lumborum. White scale bar, 1 mm, black scale bar, 200 μm. **Figure S4.** Quantitative volume analysis of calcified deposits in mdx mouse. (a) Triceps brachii muscles from left and right side of the animals were compared. Differences between left vs. right side presented as % mineralization of muscle volume were not statistically significant (Wilcoxon test (W = 0.0, number of pairs = 3), *p* > 0.999). (b) Analysis of volume of muscles replaced by calcific deposits. Percent of mineralization in triceps brachii, quadriceps femoris and spinalis pars lumborum muscles was significantly different (Kruskal-Wallis test (K-W statistic = 5.6), *p* = 0.05, *n* = 3 per each muscle group). **Figure S5.** Perfusion-based CUBIC clearing does not alter microstructure of muscle. Perfusion-based CUBIC cleared triceps brachii and quadriceps femoris muscles of mdx and C57BL/10 control animals were subjected to standard hematoxylin and eosin histological staining. Low (10×) and high (20×) magnification representative images present microstructure preservation. It has to be noted that centrally positioned nuclei, a prominent feature of dystrophy, is maintained during mdx clearing. Scale bar, 100 μm. (ZIP 12014 kb)

## Abbreviations

3DISCO: Three-dimensional imaging of solvent-cleared organs
CT: Computed tomography
CUBIC: *Clear*, Unobstructed Brain/Body Imaging Cocktails and Computational analysis
DMD: Duchenne muscular dystrophy
LSFM: Light-sheet fluorescence microscopy
MRI: Magnetic resonance imaging
PBS: Phosphate-buffered saline
PFA: Paraformaldehyde
R1: Reagent 1
SERCA: Sarco/endoplasmic reticulum Ca2+ ATPase
TOC: Tissue optical clearing
