# Supplementary figures and images for: Whole-body clearing, staining and screening of calcium deposits in the mdx mouse model of Duchenne muscular dystrophy
Source: Skelet Muscle. 2018 Jul 19;8:21. doi: 10.1186/s13395-018-0168-8 (PMC6053777; doi:10.1186/s13395-018-0168-8)

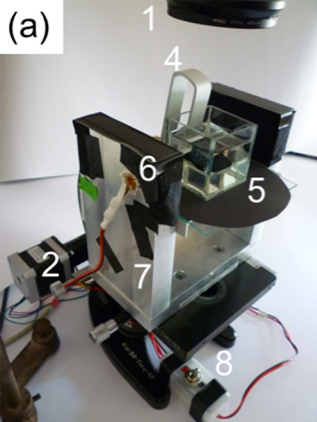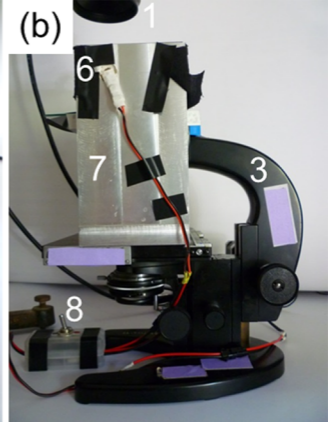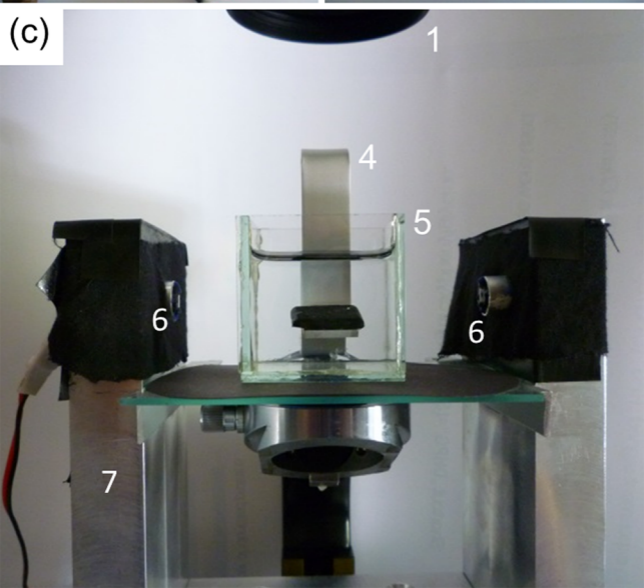

Supplement: Supplementary file 1 — Figure S1. Implementation of apparatus for macroscopic imaging using light-sheet illumination. (a) General view. (b) Side view. (c) Front view. (1) camera, (2) stepper motor, (3) z-axis line module (here light microscope body, Zeiss), (4) base holder, (5) glass container, (6) line lasers, (7) metal block, (8) laser power supply. Figure S2. Perfusion-based CUBIC cleared rat organs stained with propidium iodide. Bright field images of whole rat organs (a) intestine, (b) kidney, (c) heart, (d) cerebellum and (e) spleen. Single squares in all panels - 5 × 5 mm. This figure has been adapted from the original article “Optimized perfusion-based CUBIC protocol for the efficient whole-body clearing and imaging of rat organs” by P. Matryba et al., J Biophotonics 2017, doi./10.1002/jbio.201700248. Reproduced with permission 4358370973288. Figure S3. Comparison between optical and standard histopathology sectioning of Alizarin red S stained mdx mouse muscles. (a) Representative optical sections acquired during imaging with custom-made LSFM. Control animals present no staining-positive tissue. (b) Representative histopathology 4 μm sections observed with low and (c) high magnification. It has to be noted that histopathology images stay in line with optical sectioning, presenting petite deposits in triceps brachii and similar amount of deposits between quadriceps femoris and spinalis pars lumborum. White scale bar, 1 mm, black scale bar, 200 μm. Figure S4. Quantitative volume analysis of calcified deposits in mdx mouse. (a) Triceps brachii muscles from left and right side of the animals were compared. Differences between left vs. right side presented as % mineralization of muscle volume were not statistically significant (Wilcoxon test (W = 0.0, number of pairs = 3), p > 0.999). (b) Analysis of volume of muscles replaced by calcific deposits. Percent of mineralization in triceps brachii, quadriceps femoris and spinalis pars lumborum muscles was significantly different (Kruskal-Wa [file 13395_2018_168_MOESM1_ESM.zip › Fig. S1.pdf]

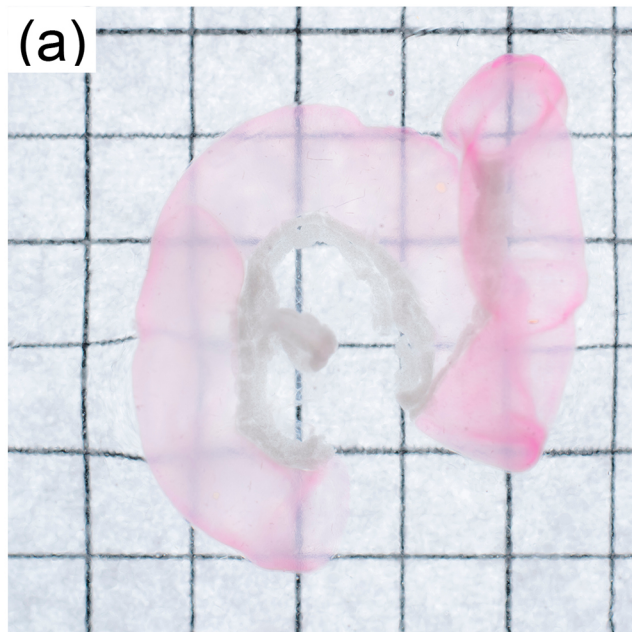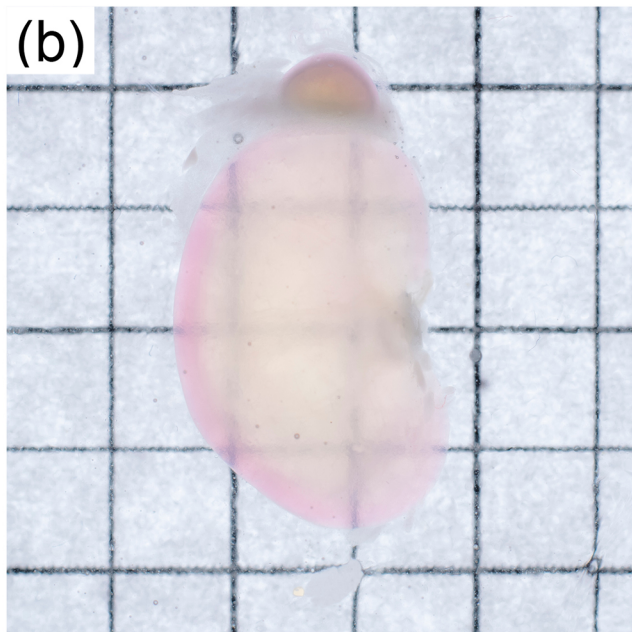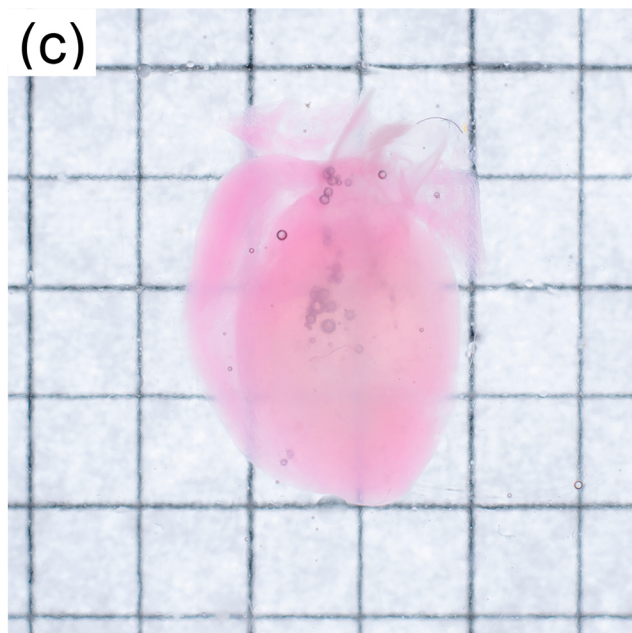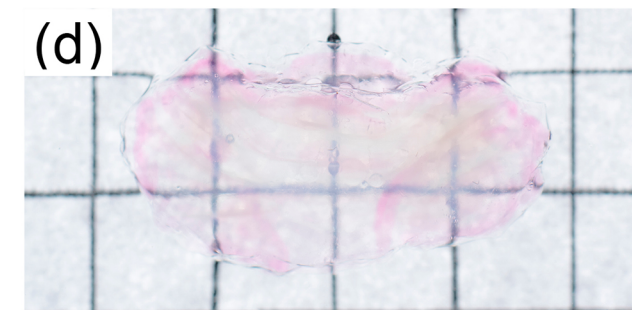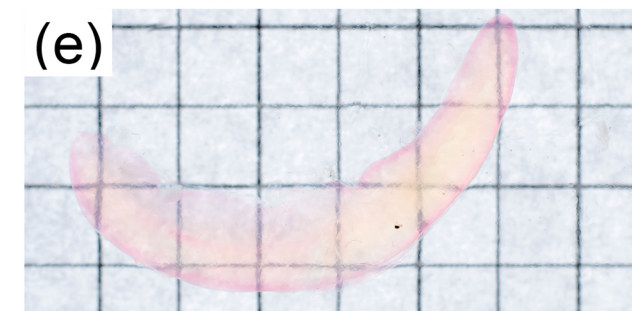

Supplement: Supplementary file 1 — Figure S1. Implementation of apparatus for macroscopic imaging using light-sheet illumination. (a) General view. (b) Side view. (c) Front view. (1) camera, (2) stepper motor, (3) z-axis line module (here light microscope body, Zeiss), (4) base holder, (5) glass container, (6) line lasers, (7) metal block, (8) laser power supply. Figure S2. Perfusion-based CUBIC cleared rat organs stained with propidium iodide. Bright field images of whole rat organs (a) intestine, (b) kidney, (c) heart, (d) cerebellum and (e) spleen. Single squares in all panels - 5 × 5 mm. This figure has been adapted from the original article “Optimized perfusion-based CUBIC protocol for the efficient whole-body clearing and imaging of rat organs” by P. Matryba et al., J Biophotonics 2017, doi./10.1002/jbio.201700248. Reproduced with permission 4358370973288. Figure S3. Comparison between optical and standard histopathology sectioning of Alizarin red S stained mdx mouse muscles. (a) Representative optical sections acquired during imaging with custom-made LSFM. Control animals present no staining-positive tissue. (b) Representative histopathology 4 μm sections observed with low and (c) high magnification. It has to be noted that histopathology images stay in line with optical sectioning, presenting petite deposits in triceps brachii and similar amount of deposits between quadriceps femoris and spinalis pars lumborum. White scale bar, 1 mm, black scale bar, 200 μm. Figure S4. Quantitative volume analysis of calcified deposits in mdx mouse. (a) Triceps brachii muscles from left and right side of the animals were compared. Differences between left vs. right side presented as % mineralization of muscle volume were not statistically significant (Wilcoxon test (W = 0.0, number of pairs = 3), p > 0.999). (b) Analysis of volume of muscles replaced by calcific deposits. Percent of mineralization in triceps brachii, quadriceps femoris and spinalis pars lumborum muscles was significantly different (Kruskal-Wa [file 13395_2018_168_MOESM1_ESM.zip › Fig. S2.pdf]

triceps brachii

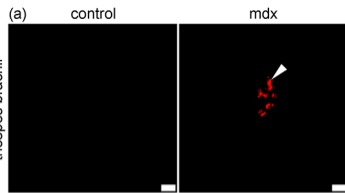

quadriceps femoris

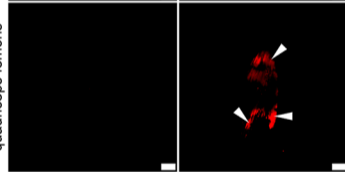

spinalis pars lumborum

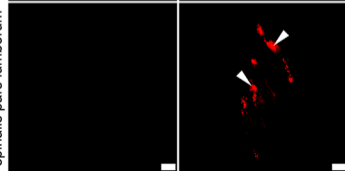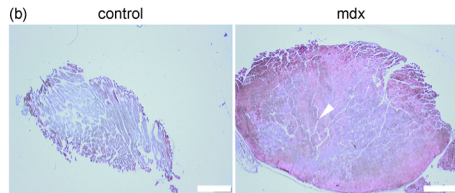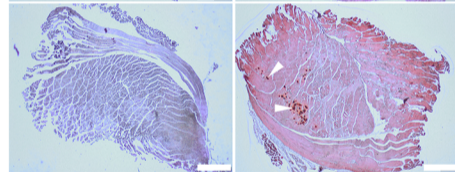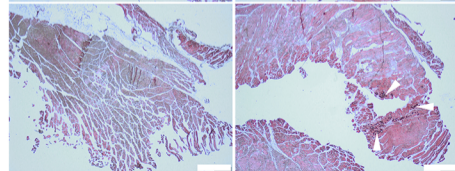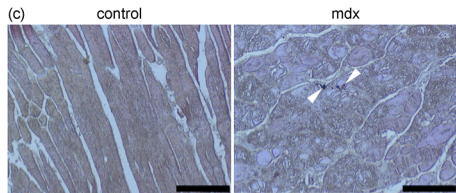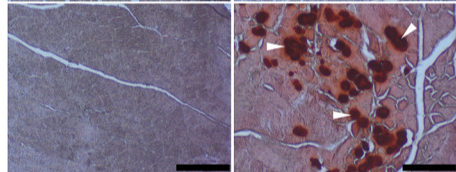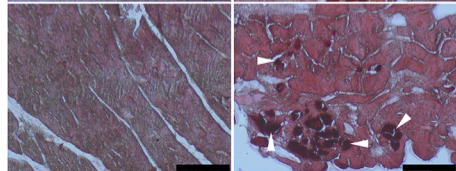

Supplement: Supplementary file 1 — Figure S1. Implementation of apparatus for macroscopic imaging using light-sheet illumination. (a) General view. (b) Side view. (c) Front view. (1) camera, (2) stepper motor, (3) z-axis line module (here light microscope body, Zeiss), (4) base holder, (5) glass container, (6) line lasers, (7) metal block, (8) laser power supply. Figure S2. Perfusion-based CUBIC cleared rat organs stained with propidium iodide. Bright field images of whole rat organs (a) intestine, (b) kidney, (c) heart, (d) cerebellum and (e) spleen. Single squares in all panels - 5 × 5 mm. This figure has been adapted from the original article “Optimized perfusion-based CUBIC protocol for the efficient whole-body clearing and imaging of rat organs” by P. Matryba et al., J Biophotonics 2017, doi./10.1002/jbio.201700248. Reproduced with permission 4358370973288. Figure S3. Comparison between optical and standard histopathology sectioning of Alizarin red S stained mdx mouse muscles. (a) Representative optical sections acquired during imaging with custom-made LSFM. Control animals present no staining-positive tissue. (b) Representative histopathology 4 μm sections observed with low and (c) high magnification. It has to be noted that histopathology images stay in line with optical sectioning, presenting petite deposits in triceps brachii and similar amount of deposits between quadriceps femoris and spinalis pars lumborum. White scale bar, 1 mm, black scale bar, 200 μm. Figure S4. Quantitative volume analysis of calcified deposits in mdx mouse. (a) Triceps brachii muscles from left and right side of the animals were compared. Differences between left vs. right side presented as % mineralization of muscle volume were not statistically significant (Wilcoxon test (W = 0.0, number of pairs = 3), p > 0.999). (b) Analysis of volume of muscles replaced by calcific deposits. Percent of mineralization in triceps brachii, quadriceps femoris and spinalis pars lumborum muscles was significantly different (Kruskal-Wa [file 13395_2018_168_MOESM1_ESM.zip › Fig. S3.pdf]

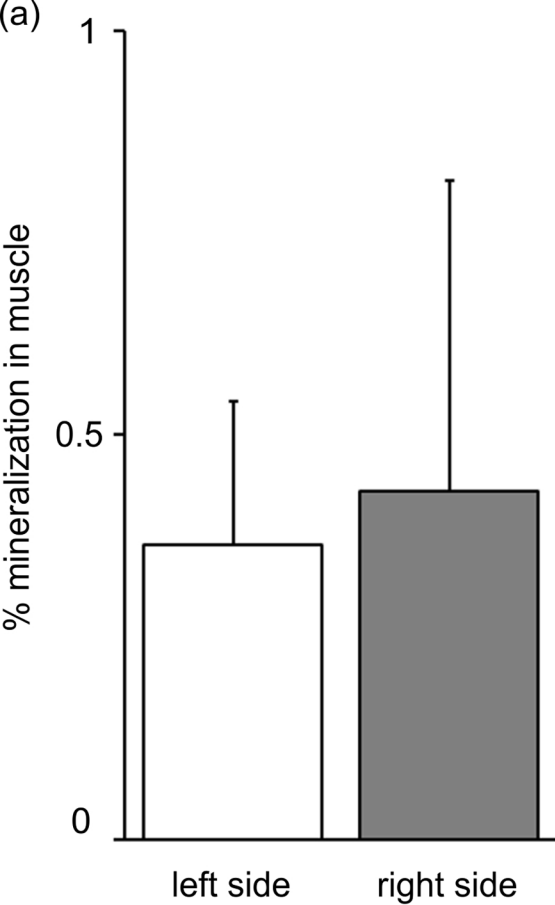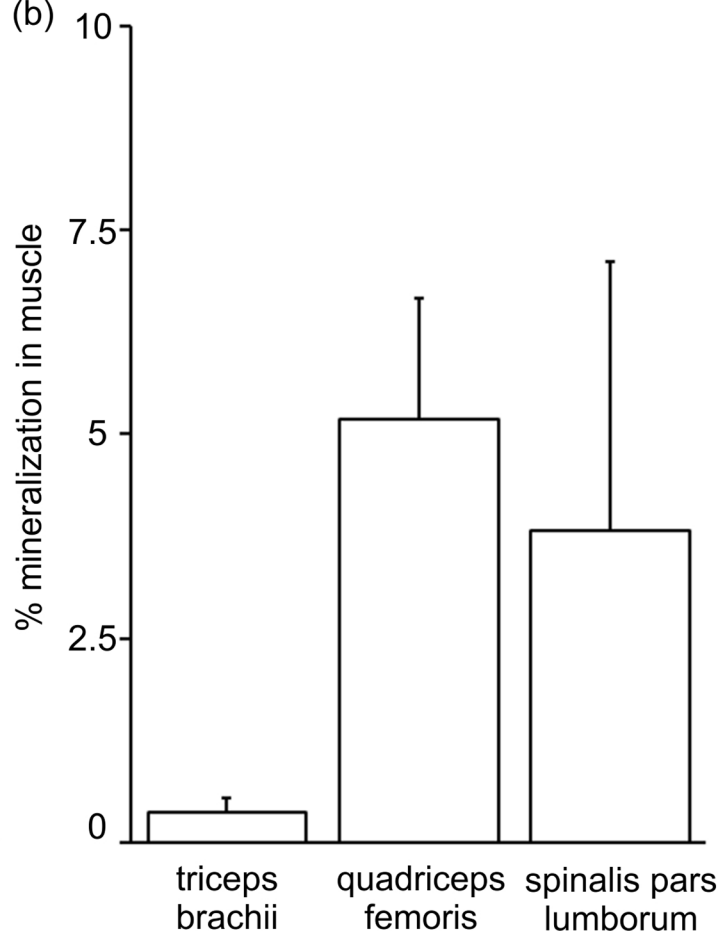

Supplement: Supplementary file 1 — Figure S1. Implementation of apparatus for macroscopic imaging using light-sheet illumination. (a) General view. (b) Side view. (c) Front view. (1) camera, (2) stepper motor, (3) z-axis line module (here light microscope body, Zeiss), (4) base holder, (5) glass container, (6) line lasers, (7) metal block, (8) laser power supply. Figure S2. Perfusion-based CUBIC cleared rat organs stained with propidium iodide. Bright field images of whole rat organs (a) intestine, (b) kidney, (c) heart, (d) cerebellum and (e) spleen. Single squares in all panels - 5 × 5 mm. This figure has been adapted from the original article “Optimized perfusion-based CUBIC protocol for the efficient whole-body clearing and imaging of rat organs” by P. Matryba et al., J Biophotonics 2017, doi./10.1002/jbio.201700248. Reproduced with permission 4358370973288. Figure S3. Comparison between optical and standard histopathology sectioning of Alizarin red S stained mdx mouse muscles. (a) Representative optical sections acquired during imaging with custom-made LSFM. Control animals present no staining-positive tissue. (b) Representative histopathology 4 μm sections observed with low and (c) high magnification. It has to be noted that histopathology images stay in line with optical sectioning, presenting petite deposits in triceps brachii and similar amount of deposits between quadriceps femoris and spinalis pars lumborum. White scale bar, 1 mm, black scale bar, 200 μm. Figure S4. Quantitative volume analysis of calcified deposits in mdx mouse. (a) Triceps brachii muscles from left and right side of the animals were compared. Differences between left vs. right side presented as % mineralization of muscle volume were not statistically significant (Wilcoxon test (W = 0.0, number of pairs = 3), p > 0.999). (b) Analysis of volume of muscles replaced by calcific deposits. Percent of mineralization in triceps brachii, quadriceps femoris and spinalis pars lumborum muscles was significantly different (Kruskal-Wa [file 13395_2018_168_MOESM1_ESM.zip › Fig. S4.pdf]

control

mdx

triceps brachii

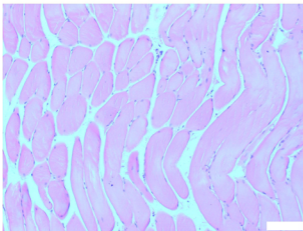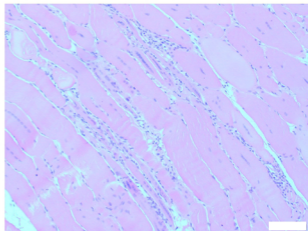

quadriceps femoris

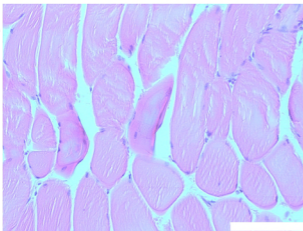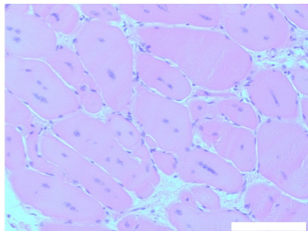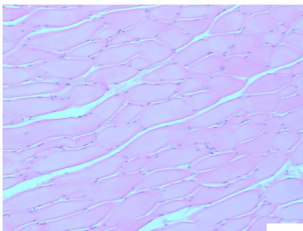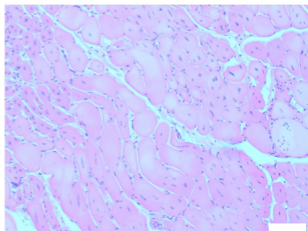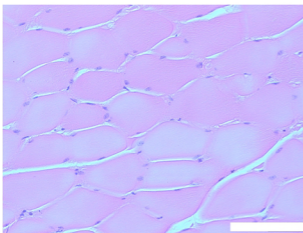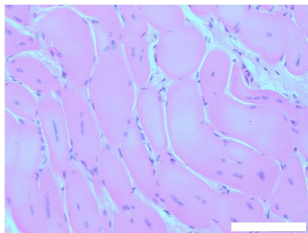

Supplement: Supplementary file 1 — Figure S1. Implementation of apparatus for macroscopic imaging using light-sheet illumination. (a) General view. (b) Side view. (c) Front view. (1) camera, (2) stepper motor, (3) z-axis line module (here light microscope body, Zeiss), (4) base holder, (5) glass container, (6) line lasers, (7) metal block, (8) laser power supply. Figure S2. Perfusion-based CUBIC cleared rat organs stained with propidium iodide. Bright field images of whole rat organs (a) intestine, (b) kidney, (c) heart, (d) cerebellum and (e) spleen. Single squares in all panels - 5 × 5 mm. This figure has been adapted from the original article “Optimized perfusion-based CUBIC protocol for the efficient whole-body clearing and imaging of rat organs” by P. Matryba et al., J Biophotonics 2017, doi./10.1002/jbio.201700248. Reproduced with permission 4358370973288. Figure S3. Comparison between optical and standard histopathology sectioning of Alizarin red S stained mdx mouse muscles. (a) Representative optical sections acquired during imaging with custom-made LSFM. Control animals present no staining-positive tissue. (b) Representative histopathology 4 μm sections observed with low and (c) high magnification. It has to be noted that histopathology images stay in line with optical sectioning, presenting petite deposits in triceps brachii and similar amount of deposits between quadriceps femoris and spinalis pars lumborum. White scale bar, 1 mm, black scale bar, 200 μm. Figure S4. Quantitative volume analysis of calcified deposits in mdx mouse. (a) Triceps brachii muscles from left and right side of the animals were compared. Differences between left vs. right side presented as % mineralization of muscle volume were not statistically significant (Wilcoxon test (W = 0.0, number of pairs = 3), p > 0.999). (b) Analysis of volume of muscles replaced by calcific deposits. Percent of mineralization in triceps brachii, quadriceps femoris and spinalis pars lumborum muscles was significantly different (Kruskal-Wa [file 13395_2018_168_MOESM1_ESM.zip › Fig. S5.pdf]
